# Development of a molecular marker for cherry crinkle leaf disease

**DOI:** 10.1186/s43897-023-00068-x

**Published:** 2023-10-08

**Authors:** Jing Wang, Xiaoming Zhang, Guohua Yan, Yu Zhou, Xuwei Duan, Chuanbao Wu, Xin Zhang, Kaichun Zhang

**Affiliations:** 1 Institute of Forestry and Pomology, Beijing Academy of Agriculture and Forestry Sciences, Beijing, 100093 P.R. China; 2 Cherry Engineering and Technical Research Center of the State Forestry and Grassland Administration, Beijing, 100093 P. R. China; 3 Key Laboratory of Biology and Genetic Improvement of Horticultural Crops (North China), Ministry of Agriculture and Rural Affairs, Beijing, China; 4 Beijing Engineering Research Center for Deciduous Fruit Trees, Beijing, China

## Abstract

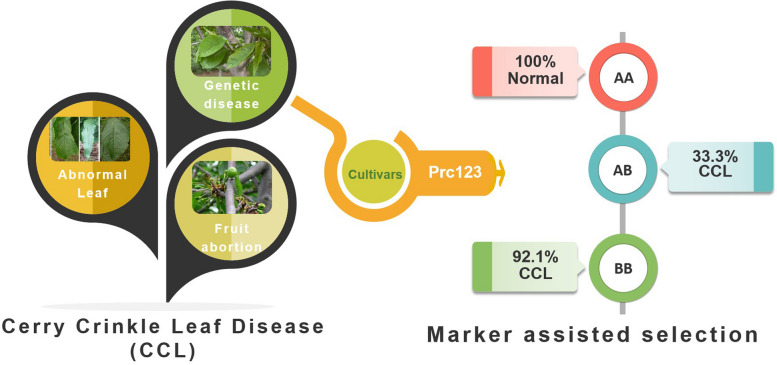

Sweet cherry (*Prunus avium L.*) is an economic fruit tree of the *Prunus* genus in the *Roaceae* family (Vignati et al. 2022). It is popular with consumers because of its early ripening in open-field, bright color, and rich nutrition. Since the 80’s, sweet cherry industry in our country has developed rapidly. At present, the production, planting area and import of sweet cherry are ranked first in the world, which is known as ‘gold planting industry’ (Zhang et al. 2020).

However, the occurrence of cherry crinkle leaf disease restricts the development of the industry. In recent years, cherry crinkle leaf disease (CCL) has been found in Shandong, Gansu and Beijing (Wang et al. 2012; 2014). CCL is mainly characterized by rough leaf surface, light color, irregular or narrow leaf edge. Fruit abortion is serious, a small number of fruits can develop and mature, but the fruit is small, deformed, with delayed maturity (Kinman 1930). In orchards with mild manifestations, the severity of CCL varied from year to year, and the location of CCL was not fixed (Helton 1962). CCL was once considered a viral disease, but grafting failed to transmit it (Kerr et al. 1956; Kerr 1963; Kinman et al. 1951; Wang et al. 2012). Classical genetic hybridization experiments have proved that CCL is a genetic disease controlled by recessive genes (Kerr 1963). It mainly occurs on sweet cherry varieties such as ‘Black Tartarian’, ‘Bing’, ‘Black Republican’ and ‘Hongdeng’ (Kinman et al. 1951; Aticinsos et al. 1959; Wang et al. 2012). CCL seriously affects the yield of cherry orchards. So, it is necessary to study the molecular markers of CCL disease, screen the existing breeding parents, to assist sweet cherry breeding.

In order to develop molecular marker of CCL, the F_1_ population of ‘Rainier × 21–21’ was used. The maternal parent ‘Rainier’ is a variety developed jointly by the Washington State Agricultural Experiment Station and the United States Department of Agriculture. Its parents are ‘Bing’ and ‘Van’. The paternal parent ‘21–21’ is a sweet cherry superiors cultivated by the Cherry Research Group of Beijing Academy of Agriculture and Forestry Sciences, which is self-crossed from ‘Stella’. No symptoms of CCL were found on ‘Rainier’ and ‘21–21’. This family had 257 individuals including 77 with CCL. CCL seedlings showed irregular leaf edge, uneven color, deep crack or rough surface, most with dwarfing symptoms (Fig. 1A, B). The ratio of CCL and normal seedling was 1:2.36 conformed to the 1:3 by Chi-square test. Perhaps, ‘Rainer’ and ‘21–21’ were both carriers of CCL. A total of 12 mixing pools were formed from leaf genomic DNA of 48 normal and 48 CCL respectively. 368 SSRs were used to detect stable difference between normal and CCL pools. In the 368 SSRs, 253 obtained the expected amplification products, accounting for 68.8%. Among them, 15 SSRs were polymorphic between the normal and the CCL pools, accounting for 5.9% of the effectively amplified SSRs. The 15 SSRs were tested by 96 individuals who made up the mixed pool. And only the genotype of Prc123 was stably correlated with the phenotype.

**Fig. 1:**
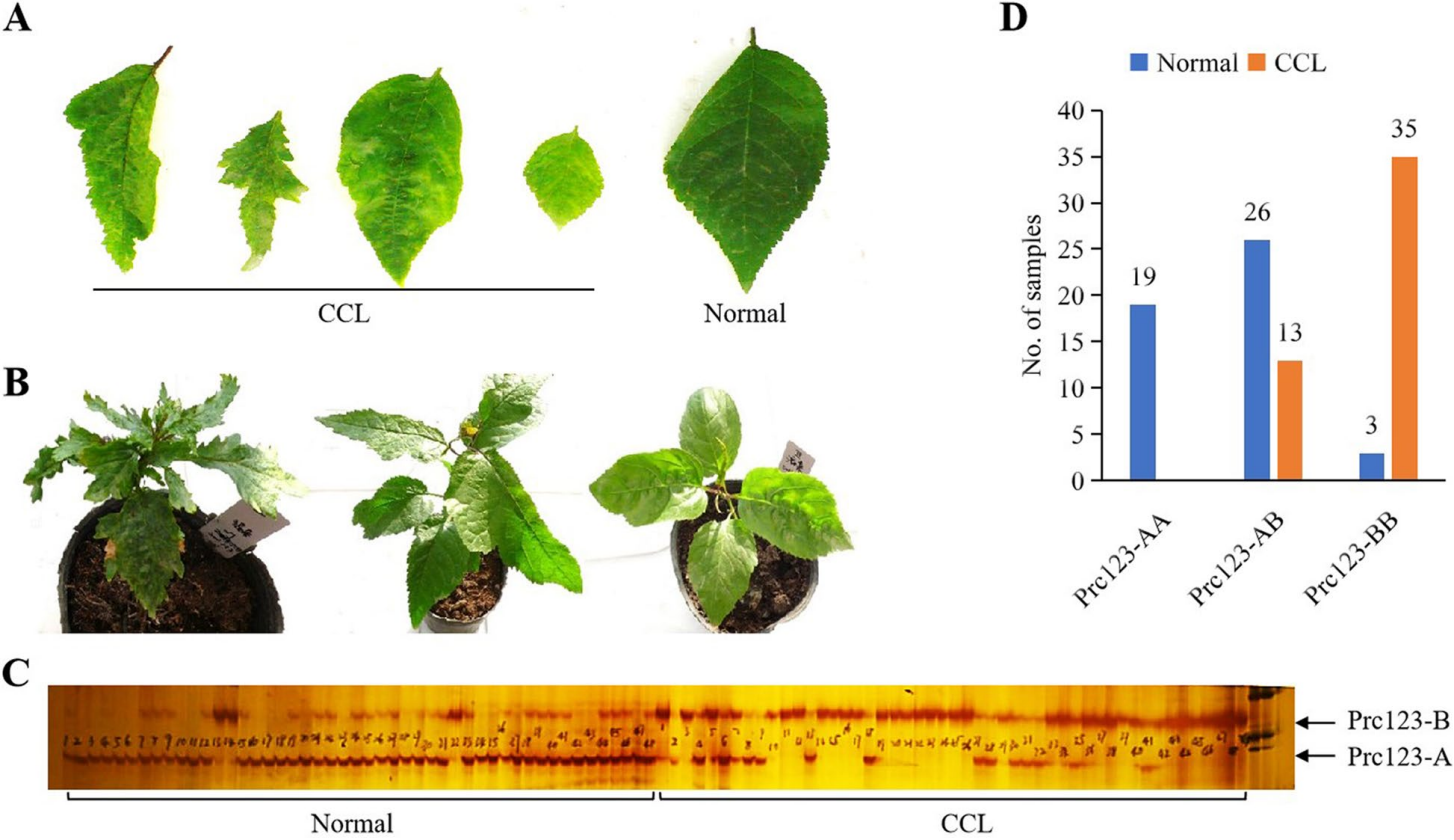
**A** Leaf symptom of CCL. **B** Whole seedling symptom of CCL. **C** The amplification result of Prc123 in 96 hybrids by using primer sequence (5 '- 3') of Prc123-F: ACCGTTTCAGCGAGAGA and Prc123-R: GCAAACCAAGCTCAGAAAG. **D** Distribution of three genotypes of Prc123 in normal and CCL individuals

Prc123 amplified two bands in sweet cherry genome, named Prc123-A and Prc123-B respectively. There were three combinations of Prc123-A and Prc123-B, namely AA, AB and BB (Fig. 1C). In normal individuals, 19 genotypes were AA, 26 were AB, and 3 were BB. Among individuals with CCL, 35 genotypes were BB and 13 were AB (Fig. 1D). Therefore, AA and AB were the main genotypes in normal individuals, and BB was the main genotype in individuals with CCL. Using Prc123 to predict the CCL, 100% Prc123-AA should be normal, 33.3% Prc123-AB genotype should be CCL and 92.1% Prc123-BB should be CCL.

In addition, 25 cultivars of sweet cherry were surveyed for genotype of Prc123, and 24 cultivars had clear amplification results. Among them, 11 cultivars were Prc123-AA, including ‘Mingzhu’, ‘Космическая’, ‘Дилемма’, ‘Van’, ‘Tieton’, ‘Caihong’, ‘PyσИНОВаЯРаННЯЯ’, ‘Zaodan’, ‘Summit’, ‘Wanhongzhu’ and ‘Sunburst’, which hadn’t been reported CCL in field. 13 cultivars were Prc123-AB type, including ‘Juhong’, ‘Rainier’, ‘Lapins’, ‘Caixia’, ‘Hongdeng’, ‘Hedelfinger’, ‘Valerij Cskalov’, ‘Burlat’, ‘Hongyan’, ‘Zaolu’, ‘Hongmi’, ‘Крупноплодная’ and ‘21–21’, in which 5 cultivars was detected CCL in field, including ‘Hedelfinger’, ‘Hongdeng’, ‘Valerij Cskalov’, ‘Hongyan’ and ‘Hongmi’. The proportion of CCL actually occurring was consistent with the proportion predicted by Prc123-AB.

To sum up, this study obtained a molecular marker of CCL, Prc123 and identified genotypes of Prc123 in 24 sweet cherry main cultivars, which can be used in preventing the CCL from spreading and molecular assisted breeding for non-CCL cultivars.

## Data Availability

The datasets used during the current study are available from the corresponding author upon reasonable request.
